# A Man Who Has Slept Five Years

**Published:** 1854-02

**Authors:** 


A Man who has Slept Five Years.—We called yesterday to see the man who has been asleep for five years, and whose case was detailed in the *Times* some weeks since. We found him in what seemed like a sound sleep. He was lying in bed, his eyes nearly closed, his respiration rather slower than usual, his breathing a little stertorious, pulse some seventy-five strokes in a minute, soft and weak. On attempting to open his eyes, he firmly closed them, and when, by force, the lids were opened, the eyes were rolled upward, so that it was impossible to see the pupils. The mouth was slightly opened; on attempting to open it wider, the jaws were instantly locked. There was a constant tremor of the eye-lids, and from the mouth there was some drivelling. His body was extremely emaciated; his arms were folded upon his breast, and any attempt to remove them was strongly resisted. The muscles seemed rigid and tense when the effort was made, and, indeed, it was impossible, without violence, to change at all the position of his limbs. Once during our stay, he drew a long breath, like a man who is about to turn in his sleep. At another time, he hitched himself up a little in bed. He was lifted up bodily and seated on the side of the bed; his head was still bent forward on his chest, his legs crooked under him at the same angle, and his arms folded as when he was lying. There was nothing to indicate that he would not retain the same position for weeks. We lifted one foot, the other came up with it. There was little or no bending at the knee or at the hip, the feet were raised only as the upper part of the body was carried backwards. He was placed standing upon the floor. It required a few moments to balance him exactly, after that he stood in the same position so long as we remained, there was nothing to indicate that he would not maintain the same posture for a month.

This certainly is a most marvellous case. There is not the slightest chance for any collusion or deception in the matter. Many of our best physicians have examined him—none, so far as we can hear, believe any deception in the case to be possible. From physicians in the western part of New York, and from men of the highest standing, we are assured that the story which is told of him is perfectly true.

His name is Cornelius Vroman, he was borne in Schoharie county, but has lived, since he was seventeen years of age, in Clarkson, Monroe county, not far from Rochester. He was a hard working man, a good worker, temperate, trusty, and, at the time when his strange sleep came on, he was working on the farm of Mr. Moses Jennings. His mother is dead, he has a father and two brothers living in Clarkson. On the 19th of June, 1848, he felt unwell enough to call in Dr. John S. Cole, who found him complaining of some pain in the stomach and in the head, for which he prescribed. After this, without becoming any more sick, his sleep each night grew longer, until at last it was found impossible to wake him. Out of this sleep he has never come, to remain wakeful for more than sixteen hours at a time, and the aggregate of all his waking hours since the seizure is not over three days. At first they were oftener, but now the waking intervals recur about every six weeks. The last time he awoke was while he was in Rochester, some ten weeks since, which gives us a hope that his waking hour now approaches, and that we may see him in his wakeful condition. When awake, he seems totally unconscious of his peculiarity, and has said some things which indicate that he remembers matters as they were before his change. They say that he straightens himself up then, and walks as limberly as others. Yet now, to handle his limbs, we fear that they must be partially anchylosed. But on this point we are not satisfactorily informed.

His diet consists principally of milk, sometimes with a little bread soaked in it. It is with some difficulty that it can be administered. The jaws must be forced open, as in tetanus, and the liquid poured in between his teeth. Once, he went without any food for five days, but his friends objected to any farther conduct of the experiment, though there was no change in his symptoms during that time. When the seizure occurred, he is said to have weighed 160 pounds, now he cannot weigh over 90 pounds. His height is six feet two inches. The secretion of the kidneys is discharged once or twice a day, it is very high colored, and not much diminished in quantity. Possibly it is from habit, possibly from some remains of consciousness, that in this matter he is subject to the wishes of his attendants. The alvine evacuations are very scanty, occurring not oftener than at intervals of from six to twenty days.

Once he was left standing for three days, there was no change in his position during that time.—*N. Y. Times.*

This individual has since died.—Ed.

